# The role of chemotherapeutic drugs in the evaluation of breast tumour response to chemotherapy using serial FDG-PET

**DOI:** 10.1186/bcr2591

**Published:** 2010-06-21

**Authors:** Michal E Schneider-Kolsky, Stewart Hart, Jane Fox, Peter Midolo, John Stuckey, Michael Hofman, Vinod Ganju

**Affiliations:** 1 Department of Medical Imaging and Radiation Sciences, Faculty of Medicine, Nursing and Health Sciences, Monash University, Clayton, Wellington Road, Victoria 3800, Australia; 2 Monash Breast Unit, Monash Medical Centre (Moorabbin Campus), Centre Road, Bentleigh 3165, Victoria, Australia; 3 Department of Medical Oncology and Clinical Haematology, Monash Medical Centre (Moorabbin Campus), Bentleigh 3165, Victoria, Australia; 4 Medical Imaging Australasia (MIA), Monash Medical Centre (Moorabbin Campus), Centre Road, Bentleigh 3165, Victoria, Australia

## Abstract

**Introduction:**

The aims of this study were to investigate whether drug sequence (docetaxel followed by anthracyclines or the drugs in reverse order) affects changes in the maximal standard uptake volume (SUV_max_) on [^18^F]flourodeoxyglucose positron emission tomography (FDG-PET) during neoadjuvant chemotherapy in women with locally advanced breast cancer.

**Methods:**

Women were randomly assigned to receive either drug sequence, and FDG-PET scans were taken at baseline, after four cycles and after eight cycles of chemotherapy. Tumour response to chemotherapy was evaluated based on histology from a surgical specimen collected upon completion of chemotherapy.

**Results:**

Sixty women were enrolled into the study. Thirty-one received docetaxel followed by anthracyclines (Arm A) and 29 received drugs in the reverse order (Arm B). Most women (83%) had ductal carcinoma and 10 women (17%) had lobular or lobular/ductal carcinoma. All but one tumour were downstaged during therapy. Overall, there was no significant difference in response between the two drug regimens. However, women in Arm B who achieved complete pathological response had mean FDG-PET SUV_max _reduction of 87.7% after four cycles, in contrast to those who had no or minor pathological response. These women recorded mean SUV_max _reductions of only 27% (*P *< 0.01). Women in Arm A showed no significant difference in SUV_max _response according to pathological response. Sensitivity, specificity, accuracy and positive and negative predictive values were highest in women in Arm B.

**Conclusions:**

Our results show that SUV_max _uptake by breast tumours during chemotherapy can be dependent on the drugs used. Care must be taken when interpreting FDG-PET in settings where patients receive varied drug protocols.

## Introduction

Positron emission tomography (PET) imaging is increasingly incorporated into the routine management of cancer therapy. The predominant tracer in use is [^18^F]flourodeoxyglucose (FDG), based on the well-known increased glucose requirement of cancer cells [1]. FDG-PET over the past few years has demonstrated promise in the assessment of breast cancer response during chemotherapy, thus facilitating clinical decisions regarding patient management and providing valuable information about ways to tailor therapy to individual patient requirement [2-6].

It has been established for over a decade now that patients who achieve a complete pathological response (cPR) in the primary breast tumour and the axillary lymph nodes have improved rates of disease-free survival [7-10]. Only 20 to 30% of patients, however, respond to treatment with cPR [11]. The majority of patients achieve a partial pathological response (pPR) to chemotherapy, and these patients are at higher risk of locoregional recurrence than those patients with a cPR [8]. It is therefore imperative to assess tumour response to treatment as early as possible in order to identify those tumours that demonstrate sensitivity to the chemotherapy regimen, to direct appropriate treatment and to spare patients unnecessary chemotherapy. Conventional imaging, such as mammography and ultrasound, provide only information on anatomical changes in tumour morphology and size. Any residual masses present after completion of chemotherapy may be composed of viable tumour cells and/or fibrotic tissue, but conventional imaging modalities are largely unable to differentiate between the different cell types. Histopathology is still required in order to accurately characterise the remaining masses.

Since tumours undergo changes in metabolism before apparent changes in size, it is important to evaluate functional changes as early as possible during treatment. Several previous studies have shown that high baseline uptake of FDG by the tumour is associated with poorer survival compared with tumours with lower uptake [12-14]. It has also been demonstrated that a significant reduction in glucose uptake on FDG-PET during or after chemotherapy can predict cPR [4,5,15,16]. The information obtained from FDG-PET studies to date further suggests that changes in glucose uptake by the tumour are independent of other, traditional, predictive breast cancer markers [17]. The impetus now is to identify chemosensitive tumours as early as possible during treatment. One study has recently demonstrated that a reduction in glucose uptake after just one cycle of neoadjuvant chemotherapy is predictive of cPR [4].

Many of the reported studies, however, investigated small samples of patients undergoing various different chemotherapeutic regimens. We still know little about the functional changes of tumours in response to specific drug regimens. Hence, we designed the present prospective study to analyse serial changes in tumours of women with locally advanced breast cancer undergoing neoadjuvant chemotherapy with docetaxel followed by anthracycline-based chemotherapy (FEC100), or of women undergoing FEC100 treatment followed by docetaxel using FDG-PET, mammography, ultrasound and clinical assessment - and to compare these changes with histological changes in sequential biopsies.

## Materials and methods

### Patient selection

Women presenting consecutively with locally advanced breast cancer TMN stage T2 to T4, N0 to N3, M0 between June 2004 and June 2008 were invited to participate in the study. The diagnosis was confirmed by core needle biopsy and histopathology in all patients prior to enrolment into the study.

The inclusion criteria were: histologically or cytologically proven invasive adenocarcinoma, age ≥ 18, Karnofsky performance status index ≥ 80%, and adequate haematology parameters, renal and hepatic function.

Patients with prior history of breast cancer or other neoplasms, with other serious medical conditions - such as congestive heart failure, significant neurological or psychiatric disorders, active uncontrolled infection, active ulcers, unstable diabetes mellitus - undergoing concurrent treatment with other experimental drugs or hormonal agents, or who were pregnant were excluded from the study.

The study was approved by the human research ethics committee at our institution and all women gave written, informed consent.

### Study design

The women were randomised using a computer-generated random number allocation to receive either four cycles of docetaxel (100 mg/m^2^), followed by four cycles of combined fluorouracil (500 mg/m^2^), epirubicin (10 0 mg/m^2^) and cyclophosphamide (500 mg/m^2^) (FEC100) (Arm 1), or the reverse order of drugs (FEC100 followed by docetaxel) (Arm 2).

At baseline, prior to commencement of chemotherapy, all women underwent a clinical examination followed by mammography, ultrasound and FDG-PET. All examinations and imaging were carried out within 4 weeks of commencement of treatment. Imaging (all three modalities) was repeated after the initial four cycles of either docetaxel or FEC100 and again upon completion of the chemotherapy. All imaging was carried out at the same clinic using the same equipment. The pathological tumour response (gold standard) was assessed after surgical resection upon completion of the treatment.

### Drug regimen

All patients received either docetaxel followed by FEC100 or the same drugs in reverse order prior to surgical resection. Each drug was delivered every 21 days for four cycles. Each subject received a total of eight cycles of chemotherapy over 24 weeks. Supportive care included antiemetics and granulocyte-stimulating factor as per American Society of Clinical Oncology guidelines.

### Clinical assessment

Tumours were measured with callipers, with the two largest diameters of the tumour recorded and multiplied. Tumour sizes at the second and third examinations were compared with baseline measurements and classified into either complete clinical response (full resolution of the palpable mass), partial resolution of the mass (> 50%) or minimal resolution or clinical progression of the mass.

### Mammography

Bidimensional tumour measurements were taken in two planes. When two or more tumours were present, the largest tumour was used to calculate the tumour size and response to treatment. The area of the tumour was calculated in each of the two projections (using the formula for an area of an ellipse) and the two values were added together. Changes in tumour size were recorded as the percentage change from baseline of the combined area measurement.

### Ultrasound measurements

Both breasts and axillas were scanned at each time point at the same site using a Phillips ATL HDI 5000 with a 12.5 MHz linear transducer. Tumours were visualised in the transverse and sagittal planes. The tumour volumes were calculated at each time point using three measurements for an ellipsoid volume [18]:(1)

When more than one tumour was present, the largest tumour was used and compared in subsequent examinations.

### FDG-PET protocol and imaging

PET scans were performed using a dual-modality Siemens Biograph 2 PET/CT scanner (PET, Siemns Medical Imaging, Knoxville, Tenessee, USA; CT, Siemens Medical Imaging, Forchheim, Bavaria, Germany), which consists of a two-row spiral CT and full-ring bismuth-germanate PET. Patients were advised to fast for at least 6 hours prior to the scan. Blood glucose concentrations were measured before the tracer (FDG) was injected. If the blood glucose level was < 12 mmol/l, the patient was given diluted contrast material orally and injected with between 8 and 10 mCi FDG via an intravenous cannula. The patient was then rested for 60 minutes prior to imaging as per our routine protocol. Variations in waiting time due to technical or workflow factors were restricted to between 45 and 75 minutes (60 ± 15 minutes).

Imaging was performed from the base of skull to just inferior of the ischial tuberosities. Images were acquired in three-dimensional mode and were reconstructed with an iterative technique using an ordered subset expectation-maximisation algorithm incorporating CT-based attenuation correction. The following formula was used for the acquisition of the standardised uptake volume (SUV) in our study:(2)

where I_suv _is the image pixel value (SUV units), I_Act _is the image pixel value (Bq/cm^3^) corrected for the start acquisition start time, dose_Tstart _is the injected dose corrected for the acquisition time, weight is the patient weight (kg), and scale is the multiplier applied to SUV values.

The patient volume of distribution is approximated by the water equivalent to the patient weight. SUV values typically range from 0 to 10 after applying a scaling factor suitable for the Syngo™ software system (Siemens Medical Imaging, Forchheim, Bavaria, Germany).

All cases were reviewed by a team of five radiologists with extensive experience in nuclear medicine, as well as a nuclear medicine physician using a Siemen's eSoft workstation to review the PET, CT and fused images. All five radiologists and the nuclear medicine physician formed a consensus agreement prior to the study to standardise measurements of SUV uptake. Regions of interest were drawn using a three-dimensional contour encompassing axial, sagittal and coronal planes traced by the eSoft software after selecting the area of increasing FDG uptake in the primary breast tumour and involved axillary nodes. The maximum standardised uptake value (SUV_max_) within this region of interest was calculated, and standardised according to body weight, dose of administered FDG and time to imaging. Partial volume correction or correction for blood sugar level was not performed. If more than one FDG avid breast lesion was identified, the lesion with the highest SUV_max _was chosen as the target lesion for serial comparison. This same procedure was performed for any axillary nodal involvement.

The metabolic response of the tumour to chemotherapy was analysed using the SUV_max _values obtained on FDG-PET during and after treatment according to the following formula:(3)

All PET readers were blinded to the clinical information, as well as the mammography and ultrasound reports of all patients. The only information provided to the readers was the reports of previous FDG-PET imaging, which were required for the mid-treatment and post-treatment reports.

### Pathologic evaluation

Biopsy samples collected at baseline and after four cycles of chemotherapy were analysed by the consultant anatomical pathologist at our institution according to standard protocols. Tumour samples were fixed in 10% formaldehyde and were embedded in paraffin. Sections were cut at 5 μm thickness and stained with H & E. The tumour type, grade and extent were reported, as well as the oestrogen receptor, progesterone receptor and HER2 status.

The pathological assessment of the final resected specimen was used for the definitive outcome measure. Patients were classified into three groups according to their pathological response. Group 1 consisted of patients who were clear of tumour in surgical specimens, including nodes (cPR); Group 2 consisted of patients with either a small residual tumour mass (≥ 80% reduction) or clear nodes (pPR); and Group 3 included patients that could not be classified into Groups 1 or 2. All pathological assessments were carried out blinded to the clinical and imaging findings.

### Statistical analysis

Comparison of demographic and tumour characteristics between the two groups of patients were carried out using the Mann-Whitney U test. Comparison of the SUV_max _tumour uptake of each group between baseline and at the midpoint or after treatment was made using the Kruskal-Wallis test. Differences in sample sizes between two groups were analysed using chi-square tests or Fisher's exact tests. Comparison of SUV_max _uptake values at each time point between the two groups of patients was made using the Mann-Whitney U test. Cutoff values ≥ 75% reduction in SUV_max _from baseline were used to assess sensitivity, specificity, positive predictive value, negative predictive value and accuracy in both treatment arms. This cutoff value was chosen to fit with the published literature and to override any minor changes in SUV measurements due to variation in technical factors. Significance was afforded when *P *< 0.05. All statistical analyses were carried out using SPSS, version 16 (SPSS Inc., Chicago, IL, USA).

## Results

Sixty-eight patients were recruited into the study. Of these patients, one withdrew from the study and seven had not completed treatment and evaluation at the time of analysis. These patients were not included in the final analysis. The final number of participants therefore included 31 patients who were randomised to receive docetaxel followed by FEC100 and 29 patients who received the same drugs in reverse order.

Tables 1 and 2 demonstrate the demographic characteristics of patients and tumour characteristics according to drug regimen used. There was no significant difference in any of the patient or tumour parameters studied between the two groups of patients. The majority of patients (50/60, 83%) presented with ductal carcinoma. Seven patients (11.6%) had lobular carcinoma and three patients (5%) had mixed ductal/lobular carcinoma. Most patients (33/60, 55%) had high-grade tumours. Thirty-seven out of 60 patients (61.6%) were oestrogen receptor-positive. Although more of these patients were randomised to receive docetaxel initially rather than FEC100 (71% compared with 52%, respectively), the difference in proportion was not significant. Similarly, the proportions of women who were progesterone receptor-positive and HER2-positive were not significantly different between the two groups. The proportion of women who underwent mastectomy or a wide local excision upon completion of chemotherapy was not statistically different between the two groups. The median (ranges) resting time prior to PET imaging was 60 minutes (45 to 75 minutes).

**Table 1:** Demographic and tumour characteristics of women undergoing neoadjuvant chemotherapy according to drug regimen

Characteristic	TAX → FEC	FEC → TAX	***P *value**^**a**^
Age	52 (37-70)	48 (30-66)	0.04^b^
Pathology type			
Ductal	25/31 (80.6)	25/29 (86.2)	1.00
Ductal/lobular	2/31 (6.5)	1/29 (3.4)	n/a
Lobular	4/31 (13.4)	3/29 (10.3)	n/a
Pathology grade			
Low	4/31 (12.9)	2/29 (6.9)	0.41
Medium	11/31 (35.5)	10/29 (34.5)	0.82
High	16/31 (51.6)	17/29 (58.6)	0.86
Oestrogen receptor status			
Negative	10/31 (32.2)	13/29 (44.8)	0.29
Positive	21/31 (67.7)	16/29 (55.2)	0.25
Progesterone receptor status			
Negative	16/31 (51.6)	18/29 (62.1)	0.60
Positive	15/31 (48.4)	11/29 (37.9)	0.33
HER2 status^c^			
Negative	17/29 (58.6)	16/29 (55.2)	1.00
Positive	12/29 (41.4)	13/29 (44.8)	1.00
Surgical procedure^d^			
Mastectomy	16/30 (53.3)	17/29 (58.6)	0.86
Wide local excision	13/30 (43.3)	12/29 (41.3)	0.84
Partial mastectomy	1/30 (3.3)	0/29 (0.0)	n/a

Demographic and tumour characteristics of women undergoing neoadjuvant chemotherapy for locally advanced breast cancer according to drug regimen (*n *= 34/group). TAX, docetaxel (Taxotere^®^); FEC, combined flourouracil-epirubicin-cyclophosphamide anthracycline-based chemotherapy; n/a, not available. Data presented as median (range) or *n/N *(%). ^a^Chi-square test. ^b^Mann-Whitney U test. ^c^Not available for two patients in Arm 1 (TAX → FEC). dOne woman in Arm 1 declined surgery.

**Table 2:** Breast tumour characteristics on mammography, ultrasound and PET at baseline according to drug regimen

	TAX → FEC	FEC → TAX	***P *value**^**a**^
Total number	31	29	
Ultrasound (volume)^b^	10,669 (702 to 169,221)	11,344 (919 to 121,428)	0.49
Mammography (volume)^c^	1,685 (0 to 7,487)	1,739 (0 to 5,749)	0.95
PET (SUV_max_)	6.76 (1.58 to 24.8)	6.26 (2.3 to 26.6)	0.98

Data presented as median (range). TAX, docetaxel (Taxotere^®^); FEC, combined flourouracil-epirubicin-cyclophosphamide anthracycline-based chemotherapy; PET, positron emission tomography; SUV_max_, maximal standard uptake volume. ^a^Mann-Whitney U test. ^b^Volume based on calculation of elliptical tumour shape. ^c^Volume based on the sum of areas in the craniocaudal and mediolateral oblique appearance.

**Figure 1:**
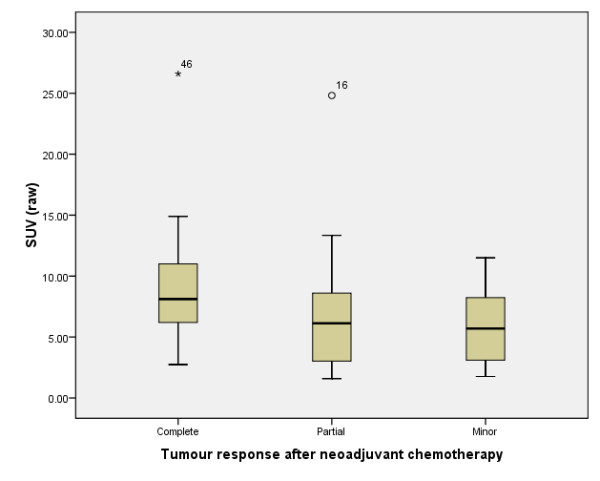
**Standard uptake values prior to neoadjuvant chemotherapy according to pathological response upon completion of chemotherapy**. The raw standardised uptake volume (SUV) values are based on those provided by the baseline [^18^F]flourodeoxyglucose-positron emission tomography scan. Data depict the median, interquartile ranges and total ranges (*N*/complete = 14, *N*/partial = 18, *N*/minor = 28).

Tumour size on mammography and ultrasound and the SUV uptake on FDG-PET were not significantly different between the two groups of patients at baseline (Table 2). The median (range) SUV_max _uptake for all women was 6.5 (1.58 to 26.6). Higher baseline SUV_max _uptake was associated with higher rates of final pathological response, although statistical significance was not reached (Figure 1).

The numbers of tumours that were classified as cPR, as pPR and as pathological response not included in cPR or pPR are presented in Table 3. All but one tumour were downstaged during neoadjuvant chemotherapy. Overall, 23% of tumours (14/60) were classified as having undergone a complete pathological response. Thirty per cent of tumours (18/60) underwent a partial response, and the other tumours (28/60 (47%) recorded only minor or no changes during chemotherapy. There was no significant difference in tumour response between the two drug regimens (*P *> 0.05).

**Table 3:** Number of tumours classified as complete, partial or minor pathological response according to drug regimen

Response	TAX → FEC	FEC → TAX	Total (all women)
cPR	6/31 (19)	8/29 (28)	14/60 (23)
pPR	9/31 (29)	9/29 (31)	18/60 (30)
mPR	16/31 (52)	12/29 (41)	28/60 (47)

Number of tumours classified as a complete pathological response (cPR), partial pathological response (pPR) or minor pathological response (mPR) after completion of neoadjuvant chemotherapy according to drug regimen. Data presented as *n/N *(%). TAX, docetaxel (Taxotere^®^); FEC, combined flourouracil-epirubicin-cyclophosphamide anthracycline-based chemotherapy.

**Figure 2:**
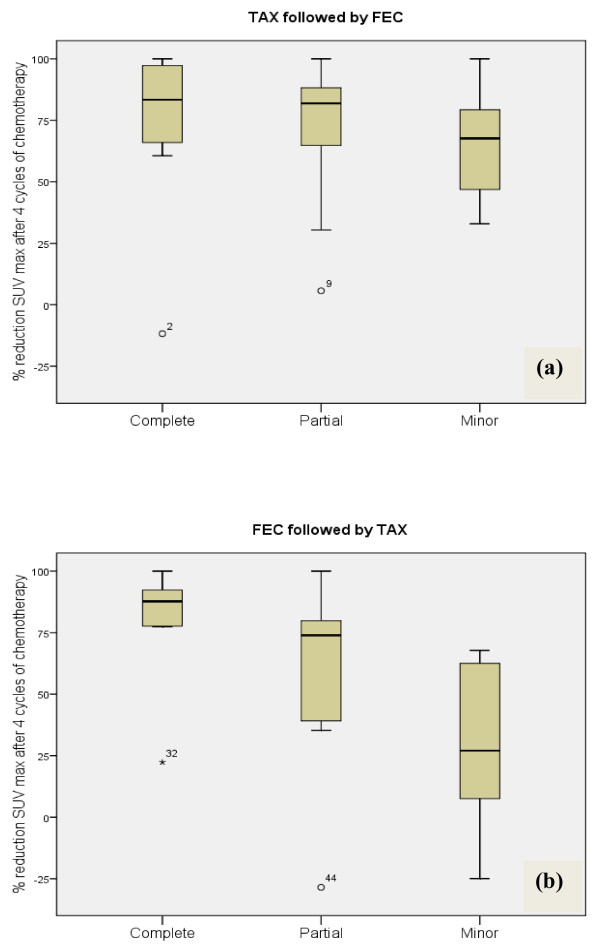
**Reduction in maximal standardised uptake volume after four cycles of neoadjuvant chemotherapy**. Percentage reduction in maximal standardised uptake volume (SUV_max_) after four cycles of neoadjuvant chemotherapy according to pathological response obtained upon completion of chemotherapy. **(a) **Reductions in SUV_max _after four cycles of docetaxel (Taxotere^®^) (TAX). **(b) **Reductions in SUV_max _after four cycles of anthracycline-based fluorouracil, epirubicin and cyclophosphamide (FEC). Data depict the median, interquartile ranges and total ranges. (a) *N*/complete = 6, *N*/partial = 9, *N*/minor = 16. (b) *N*/complete = 8, *N*/partial = 9, *N*/minor = 12.

**Figure 3:**
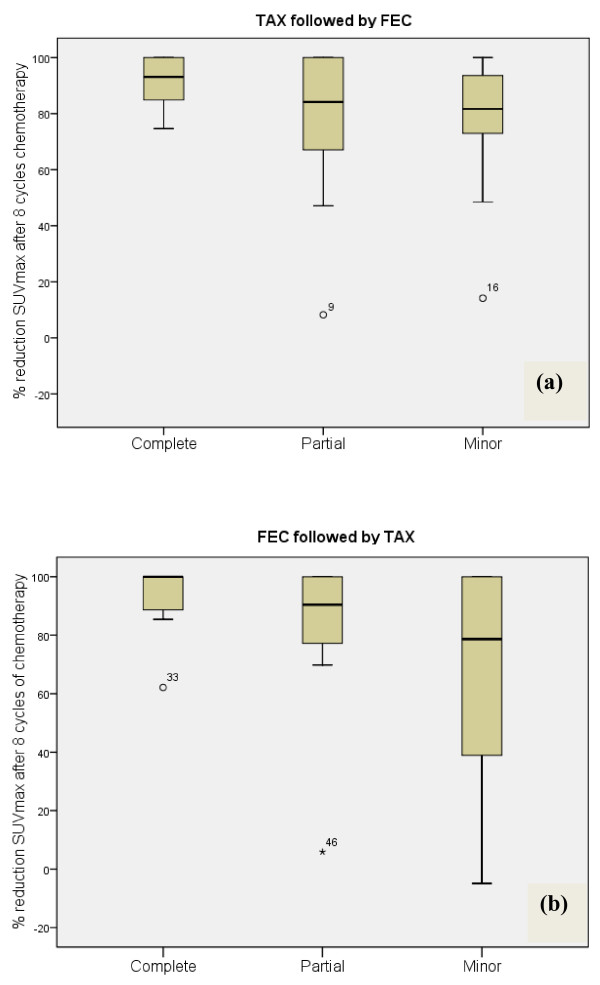
**Reduction in maximal standardised uptake volume after eight cycles of neoadjuvant chemotherapy**. Percentage reduction in maximal standardised uptake volume (SUV_max_) from baseline after eight cycles of neoadjuvant chemotherapy according to pathological response obtained upon completion of chemotherapy. **(a) **Reductions in SUV_max _after four cycles with docetaxel (Taxotere^®^) (TAX) followed by four cycles with anthracycline-based fluorouracil, epirubicin and cyclophosphamide (FEC). **(b) **Reductions in SUV_max _after four cycles with FEC followed by four cycles with TAX. Data depict the median, interquartile ranges and total ranges. (a) *N*/complete = 6, *N*/partial = 9, *N*/minor = 16. (b) N *N*/complete = 7, *N*/partial = 9, *N*/minor = 12).

Table 4 demonstrates the reduction in tumour volumes after four and eight cycles of chemotherapy as observed on mammography, ultrasound and FDG-PET according to the drug given. There was no statistically significant difference between the two groups of patients when comparing tumour volume reduction or SUV_max _uptake on either imaging modality. The only significant difference observed was associated with SUV_max _uptake after four cycles of chemotherapy with anthracyclines. In this group of women, minor or no final pathological response was associated with minimal reduction of SUV_max _uptake compared with women receiving docetaxel (*P *= 0.01). These outcomes are also highlighted in Figure 2. Women receiving FEC100 first who achieved a cPR had a median reduction of SUV_max _of 87.7% from baseline (Figure 2a). In contrast, those on the same drug whose tumour recorded no or only minor pathological response had a median reduction of only 27% (Figure 2b). These data are significantly different from those by women who received docetaxel initially (*P *= 0.01). This group had no significant difference in the median SUV_max _reduction according to pathological response. The percentage reduction in SUV_max _uptake after all eight cycles is shown in Figure 3. There were no significant differences between the two drug sequences in terms of pathological response (*P *> 0.05).

**Table 4:** Reduction in tumour volumes after four and eight cycles of chemotherapy according to drug regimen

	TAX → FEC	FEC → TAX	***P *value**^**a**^
Total number	31	29	
SUV_max _reduction after four cycles			
Ultrasound	80.6 (-271 to 100)	76.0 (-195 to 100)	0.89
Mammography	54.4 (-100 to 100)	50.7 (-337 to 100)	0.36
PET	75.0 (-11 to 100)	62.7 (-29 to 100)	0.16
According to final pathological response			
Complete response	77.3 (-12 to 100)	82.8 (22 to 100)	0.81
Partial response	81.8 (6 to 100)	73.9 (-28 to 100)	0.79
Minor/no response	67.0 (33 to 100)	27.0 (-25 to 68)	0.01
SUV_max _reduction after eight cycles			
Ultrasound	91.9 (-5.6 to 100)	92.5 (-155 to 100)	0.95
Mammography	77.3 (-119 to 100)	77.5 (-84 to 100)	0.74
PET	84.1 (8.2 to 100)	91.1 (-4.8 to 100)	0.36

Percentage reduction in tumour volumes on mammography and ultrasound, and maximal standard uptake volumes (SUV_max_) on [^18^F]flourodeoxyglucose positron emission tomography (PET) after four and eight cycles of chemotherapy according to drug regimen. Data presented as median (range). TAX, docetaxel (Taxotere^®^); FEC, combined flourouracil-epirubicin-cyclophosphamide anthracycline-based chemotherapy. ^a^Mann-Whitney U test.

The sensitivity, specificity, positive predictive value, negative predictive value and accuracy rates for both treatment arms are presented in Table 5. Highest sensitivity (0.87) was obtained for SUV_max _reduction after chemotherapy with FEC100 (Arm B). In contrast, sensitivity was 0.83 for women in Arm A. Specificity and overall accuracy was best among women who received Arm B (0.76 and 0.79, respectively). Similarly, both positive and negative predictive values scored highest in this group of women (0.58 and 0.94, respectively).

**Table 5:** Sensitivities, specificities, positive and negative predictive values and accuracy rates after four cycles of chemotherapy

	All women	TAX → FEC	FEC → TAX
Total number	60	31	29
Sensitivity	0.78	0.66	0.87
Specificity	0.60	0.48	0.76
Positive predictive value	0.37	0.23	0.58
Negative predictive value	0.90	0.85	0.94
Accuracy	0.65	0.51	0.79

Sensitivities, specificities, positive and negative predictive values and accuracy rates for [^18^F]flourodeoxyglucose positron emission tomography based on a 75% reduction of maximal standard uptake volume after four cycles of chemotherapy. TAX, docetaxel (Taxotere^®^); FEC, combined flourouracil-epirubicin-cyclophosphamide anthracycline-based chemotherapy.

The proportion of women who achieved SUV_max _reduction ≥ 75% after four cycles of chemotherapy in either treatment arm was also compared according to nodal status at surgery (Table 6). There was no difference between the proportion of women with ≥ 75% reduction of SUV_max _in either Arm A or Arm B (*P *= 0.63). Overall, women with ≥ 75% reduction of SUV_max _after four cycles were more likely to have negative nodes at surgery (*P *= 0.13). There was no statistical difference between the proportion of women with negative nodes who were in either Arm A or Arm B (*P *= 0.9). Among those with positive nodes, however, the majority were women who had received docetaxel rather than FEC100 initially (90% versus 10%, respectively; *P *= 0.001).

**Table 6:** Response of women to neoadjuvant chemotherapy according to final nodal status

	All women	TAX → FEC	FEC → TAX	***P *value**^**a**^
≥ 75% reduction in SUV_max_	28/57 (49.1)	17/28 (60.7)	11/28 (39.3)	0.63
Node-negative	18/28 (64.2)	8/18 (44.4)	10/18 (55.5)	0.9
Node-positive	10/28 (35.7)	9/10 (90.0)	1/10 (10.0)	0.001

Response of women to neoadjuvant chemotherapy according to final nodal status (positive or negative nodes at surgery). Women were stratified according to drug sequence and > 75% reduction in maximal standard uptake volume (SUV_max_) of [^18^F]flourodeoxyglucose after four cycles. Data presented as *n/N *(%) (total number = 57). TAX, docetaxel (Taxotere^®^); FEC, combined flourouracil-epirubicin-cyclophosphamide anthracycline-based chemotherapy. ^a^Chi-square test (comparison of the two treatment arms).

The histological cancer category (ductal, *n *= 50 vs. lobular or lobular/ductal, *n *= 10) did not influence the baseline SUV_max _uptake (*P *= 0.48) or that recorded at the halfway point or at the end of treatment (*P *= 0.96 and *P *= 0.51, respectively; Table 7). Of the 10 women with lobular or lobular/ductal cancer, seven (70%) had residual nodal disease upon completion. This observation compares with 26/50 (52%) of women with ductal cancer (*P *= 0.4).

**Table 7:** PET SUV_max _at baseline and after four cycles of chemotherapy according to histological cancer category

SUV_max_	Ductal (*n *= 50)	Lobular/lobular-ductal (*n *= 10)	***P *value**^**a**^
Baseline	6.96 (1.58 to 26.6)	6.42 (1.76 to 10.3)	0.48
After four cycles	1.85 (0 to 12.3)	1.87 (0 to 8.3)	0.96
After eight cycles	1.13 (0 to 8.7)	0.25 (0 to 5.6)	0.51

Positron emission tomography (PET) maximal standard uptake volume (SUV_max_) at baseline and after four cycles of chemotherapy (> 75% reduction in SUV_max_) according to histological cancer category. Data presented as median (range) (total number = 60). ^a^Mann-Whitney U test.

## Discussion

Women who present with locally advanced breast cancer are today routinely managed using neoadjuvant chemotherapy, with the aim of downstaging the primary tumour before surgery. The most commonly used chemotherapeutic drug in this setting is docetaxel. Sequential anthracycline-based chemotherapeutic drugs are increasingly used but little information is so far available on the sensitivity of the tumours to these drug regimens during treatment. The standard sequence of drugs for adjuvant therapy has to date involved anthracyclines followed by taxanes, but phase II trials have recently suggested that the reverse sequence may improve efficacy and lower toxicity [19]. In our neoadjuvant setting, there was no significant difference in the efficacy between the two drug regimens. In fact, slightly more women reported a complete pathological response when treated first with FEC100 compared with docetaxel (27% versus 19%, *P *= 0.46).

As expected, mammographic and ultrasound measurements of the breast tumours did not prove useful predictors of tumour response. These traditional imaging modalities utilise tumour size as a measure of tumour cell kill. Such criteria have been used for over 50 years but their clinical usefulness remains limited. Functional imaging has more recently moved to the forefront of these investigations. PET with or without co-registration of CT using the radiolabelled glucose analogue FDG shows promise for diagnosis, for staging and as a predictive marker of tumour response for a range of tumours, because changes in glucose metabolism occur prior to any significant changes in tumour size [20].

Results in breast cancer patients to date have shown that SUV on FDG-PET after only one or two cycles of chemotherapy can be used to measure early response, with recorded sensitivities of between 61 and 100% and specificities of between 75 and 100% [15,21-24]. Analysis of these outcomes is difficult, however, since the disease stage, the definition of response, the drugs and sequence of drugs used, the calculation of FDG uptake and the cutoff points used to evaluate sensitivities and specificities varied among the patient cohorts. Further, most of these studies evaluated small populations (< 25 participants) - with the exception of the study by Rousseau and colleagues that evaluated 64 patients with stage II and III breast cancer [22], and the study by Smith and colleagues who investigated 31 women with large (> 3 cm) breast tumours [25].

One aspect of these studies that has not received a lot of attention is the SUV uptake in response to the drug regimen used. We hypothesised that the drug regimen may significantly affect the degree of reduction observed on serial PET scans because different drugs target different biological actions in the cancer cells. Most studies to date have all evaluated FDG-PET in cohorts of women who received various drugs with a range of dosage and sequences. It is thus difficult to assess the true usefulness of PET as a predictor of tumour response to treatment. In general, however, the literature has demonstrated that tumours with high levels of FDG uptake at baseline are more likely to achieve a complete pathological response [14,22,24,25]. These findings were confirmed in our study.

Women in our cohort who recorded SUV_max _reductions > 75% after four cycles of chemotherapy were more likely to achieve a cPR of the tumour. Further, tumours with SUV_max _reductions < 30% after four cycles of FEC100 were more likely to remain insensitive to the treatment regardless of the subsequent use of docetaxel. Sensitivity, specificity, positive and negative predictive values, as well as overall accuracy were highest (when the 75% cutoff value was used) among women receiving FEC100 initially. Berriolo-Riedinger and colleagues achieved sensitivities and specificities of 0.91 and 0.86, respectively, after one treatment cycle [4]. In their study, however, women received four different treatment protocols.

Sensitivity of 0.77 and specificity of 0.80 were achieved in a study of 50 women with very large breast tumours (mean size 4.3 cm) who had FDG-PET before and after two cycles of chemotherapy with epirubicin and cyclophosphamide and, if axillary nodes were positive, additional taxanes [24]. Rousseau and colleagues achieved sensitivities and specificities of 0.61 and 0.96, respectively, in their cohort of women using the 60% cutoff value after two cycles [22]. Our comparative values (when calculating a 60% cutoff value) after four cycles are 0.87 and 0.56 in women receiving FEC100 and are 0.83 and 0.32 in women receiving docetaxel. These values are similar to those recently reported by Jung and colleagues [26]. In their study, 66 women were divided into two groups, with 31 women receiving docetaxel plus capecitabine and the other 31 women receiving doxorubicin plus cyclophosphamide. FDG-PET was carried out before and after four cycles of chemotherapy. The authors used an 84.8% cutoff value to achieve sensitivity and specificity values of 70% and 69.6%, respectively [26]. The patients were not analysed according to drugs used, but were evaluated as a whole group.

In an earlier study, patients receiving cyclophosphamide, doxorubicin, vincristine and prednisolone with or without docetaxel were scanned using FGD-PET after one cycle and four cycles of treatment [25]. There was no difference in the SUV_max _reduction between the two treatment regimens used at either time point. Using a 20% reduction in SUV as the cutoff value, this study recorded sensitivity and specificity values of 0.90 and 0.74, respectively [25]. The patients included in that study had locally advanced cancer > 3 cm, which may have contributed to the relatively high sensitivity achieved. In our study, patients were included if their cancer was > 2 cm in size. Our less restrictive inclusion criteria may hence have resulted in the lower sensitivity and specificity values since the sensitivity of FDG-PET is known to be lower when the tumours are smaller [17].

It is interesting to compare the different drug-induced tumour responses observed on PET. The overall reduction in tumour volume was similar in all imaging modalities investigated regardless of the drugs used (Table 3). The bulk of tumour reduction occurred during the first four cycles. Women who received FEC100 and who had a median SUV_max _reduction of only 27% or less, however, were significantly more likely to have treatment-resistant tumours (*P *= 0.01). In contrast, tumours of women who had received docetaxel could not be easily stratified. Our results further show that women who reported reductions of SUV_max _uptake ≥ 75% halfway through treatment were significantly more likely to have positive nodes upon completion of chemotherapy if they had received docetaxel initially (90% vs. 10%) (Table 5). This association has not previously been reported. Since nodal status is an important prognostic indicator of overall survival and recurrence, further investigations in larger cohorts of women with adequate follow up of at least 5 years are required.

Our results demonstrate that the usefulness of PET in predicting tumour response can be influenced by the type of drugs used. This is an important consideration in the evaluation of PET during chemotherapy. All previous studies, with the exception of that by Smith and colleagues [25], evaluated groups of patients with mixed drug protocols. Given the various modes of actions of these drugs on tumour cells, it is not surprising that statistically significant differences in metabolic activity of the tumours may be observed on PET once therapy is initiated. The mechanisms of drug responsiveness/resistance are not fully understood. Docetaxel and FEC100 affect multiple pathways in tumour signalling and growth. Docetaxel disrupts microtubules, enhances apoptosis and affects angiogenesis [27,28], while FEC100 interferes with the synthesis of DNA and reduces mitotic and metabolic activity. Today, most combination neoadjuvant chemotherapies involve docetaxel with anthracyclines since this combination improves the pathological response rates in primary breast cancers and results in improved disease-free survival and overall survival, at least in the adjuvant setting [28-30]. Nevertheless, randomised prospective studies are needed in order to clarify how each drug protocol affects tumour glucose metabolism and the appearance/activity on serial FDG-PET studies.

SUV uptake values can be influenced by a range of factors. Core or fine-needle aspiration biopsies can potentially cause local inflammatory reaction, which could affect SUV uptake and mask true tumour uptake. This can impact on subsequent results and interpretation of the SUV values reported. In our study, the initial (baseline) PET studies were carried out at least 10 to 14 days after the diagnostic core or fine-needle aspiration took place, and are therefore unlikely to significantly affect the SUV uptake recorded for the baseline imaging. Once recruited, all women were biopsied only after the PET imaging took place in order to avoid influencing SUV measurements at the mid-treatment assessment. The final PET imaging took place before surgical intervention and hence did not have any impact on the third PET imaging report.

Another potential influence on SUV measurements is related to the variations of resting times that were permitted during the study. The protocol in our centre stipulates a 60 minute resting time prior to PET imaging. This was adhered to in the great majority of patients. On occasion, however, technical issues or delays in workflow resulted in fluctuations of the resting time. This was kept to a minimum whenever possible (variations of ± 15 minutes were permitted). Other factors potentially resulting in SUV variation or inaccuracy include partial volume effects (in which there is underestimation in small tumours), patient movement between PET and CT, including respiratory motion, and variation in FDG uptake time between initial and restaging PET scans. Whilst variation in technique may impact on the SUV measurements, it is very unlikely that a marked reduction in SUV (75%) will occur purely due to technical factors. Finally, we chose to report SUV_max _values rather than SUV mean values because these are the values that are routinely reported to the treating oncologists and also to facilitate comparison of our results with those reported in the literature [22-24,26].

Our study assessed tumour response after four cycles of chemotherapy. Recent studies have shown that optimal response evaluation exists as early as after two cycles [22,23]. It would have been useful to have carried out this earlier scan in our cohort of patients and to compare the sensitivity and specificity with other published studies. This should be investigated in future studies in order to obtain drug-specific response rates at a stage during treatment when patients are not exposed to several cycles of toxic and potentially ineffective therapy. Our study was also limited by the heterogeneity of the breast tumours in our cohort of women. This heterogeneity possibly contributed to the large variation of SUV_max _values observed at baseline among our patient cohort and may be reflective of the variable biological factors inherent to breast cancer.

We have previously observed a correlation between PET SUV and a 12-gene signature [31]. Despite the biological diversity of breast cancers in our cohort of women, tumour type (ductal versus lobular) did not appear to influence SUV_max _uptake at any stage of treatment (Table 6). There was no association between residual nodal disease and PET response according to histological types of cancer. Nevertheless, given the small number of women with lobular or lobular/ductal breast cancer in our study (*n *= 10), the next series of studies should investigate the relationship between various histological breast cancer types, drugs and SUV uptake on FDG-PET in a larger cohort of women. Such information will provide clinicians with better tools to direct individualised patient management and predict treatment responses early on during therapy.

## Conclusions

Our results have shown that SUV_max _uptake by breast tumours on FDG-PET during neoadjuvant chemotherapy may be dependent on the drug regimen used. The predictive value of FDG-PET appears to be more useful in patients treated initially with anthracycline-based chemotherapy rather than taxane-based chemotherapy. Care must be taken when evaluating FDG-PET in settings where patients receive varied drug protocols. More studies are needed to clarify and standardise the relationships between tumour type, chemotherapeutic drugs and SUV uptake on serial FDG-PET imaging.

## Abbreviations

cPR: complete pathological response; CT: computed tomography; FDG: [^18^F]flourodeoxyglucose; FEC100: anthracycline-based fluorouracil, epirubicin and cyclophosphamide; H & E: haematoxylin and eosin; PET: positron emission tomography; pPR: partial pathological response; SUV_max_: maximal standardised uptake volume.

## Competing interests

The authors declare that they have no competing interests.

## Authors' contributions

MES-K carried out the data analysis, some of the data collection and wrote the manuscript. SH and JF carried out the surgical procedures (wide local excision and mastectomy) and edited the manuscript. PM carried out the data collection and edited the manuscript. JS and MH carried out the FDG-PET studies and interpreted the results. VG devised the study and co-wrote the manuscript.
